# Differential Relationship between Microstructural Integrity in White Matter Tracts and Motor Recovery following Stroke Based on Brain-Derived Neurotrophic Factor Genotype

**DOI:** 10.1155/2020/5742421

**Published:** 2020-09-22

**Authors:** Eunhee Park, Jungsoo Lee, Won Hyuk Chang, Ahee Lee, Friedhelm C. Hummel, Yun-Hee Kim

**Affiliations:** ^1^Department of Rehabilitation Medicine, Kyungpook National University Hospital, Daegu, Republic of Korea; ^2^Department of Rehabilitation Medicine, School of Medicine, Kyungpook National University, Daegu, Republic of Korea; ^3^Department of Physical and Rehabilitation Medicine, Samsung Medical Center, Sungkyunkwan University School of Medicine, Seoul, Republic of Korea; ^4^Center for Prevention and Rehabilitation, Heart Vascular Stroke Institute, Samsung Medical Center, Seoul, Republic of Korea; ^5^Department of Health Sciences & Technology, Department of Medical Device Management & Research, Department of Digital Health, SAIHST, Sungkyunkwan University, Seoul, Republic of Korea; ^6^Defitech Chair of Clinical Neuroengineering, Center for Neuroprosthetics (CNP) and Brain Mind Institute (BMI), Swiss Federal Institute of Technology (EPFL), 1202 Geneva, Switzerland; ^7^Defitech Chair of Clinical Neuroengineering, Center for Neuroprosthetics (CNP) and Brain Mind Institute (BMI), Swiss Federal Institute of Technology (EPFL Valais), Clinique Romande de Réadaptation, 1951 Sion, Switzerland; ^8^Clinical Neuroscience, University of Geneva Medical School, 1202 Geneva, Switzerland

## Abstract

**Objective:**

The relationship between white matter integrity and the brain-derived neurotrophic factor (*BDNF*) genotype and its effects on motor recovery after stroke are poorly understood. We investigated the values of fractional anisotropy (FA) in the corticospinal tract (CST), the intrahemispheric connection from the primary motor cortex to the ventral premotor cortex (M1PMv), and the interhemispheric connection via the corpus callosum (CC) in patients with the *BDNF* genotype from the acute to the subacute phase after stroke.

**Methods:**

The Fugl-Meyer assessment, upper extremity (FMA-UE), and tract-related FA were assessed at 2 weeks (T1) and 3 months (T2) after stroke using diffusion tensor imaging (DTI). Fifty-eight patients diagnosed with ischemic stroke were classified according to the *BDNF* genotype into a Val (valine homozygotes) or Met (methionine heterozygotes and homozygotes) group.

**Results:**

The Val group exhibited a larger reduction of FA values in the ipsilesional M1PMv than the Met group from T1 to T2. The FMA-UE at T2 was negatively correlated with FA of the contralesional M1PMv at T2 in the Val group but was positively correlated with FA of the ipsilesional CST and CC at T2 in the Met group.

**Conclusions:**

The integrity of the intra- and interhemispheric connections might be related to different processes of motor recovery dependent on the *BDNF* genotype. Thus, the *BDNF* genotype may need to be considered as a factor influencing neuroplasticity and functional recovery in patients with stroke. This trial is registered with http://www.clinicaltrials.gov: NCT03647787.

## 1. Introduction

Stroke is the most common cause of adult disability [1]. Motor impairment is a critical disability after stroke, and recovery of motor function is crucial for restoring independence in daily life [2]. As a key aspect of precision medicine, accurate prediction of motor recovery is needed to optimize rehabilitation goals and strategies for individual patients with stroke. However, the determination of prognostic factors associated with upper limb motor recovery poses a challenge for clinicians [3–5]. Previous studies have reported several clinical factors that can be used to predict upper limb motor recovery after stroke [1–6]. One study reported that age, severity of motor impairment at baseline, and presence or absence of a response to motor evoked potential (MEP) were significant independent predictors of upper extremity motor function in patients with stroke [5]. Another study has demonstrated a proportional recovery rule for motor function [4]; however, it is limited by mathematical bias [7] and does not apply to all patients with stroke, especially those with severe impairment.

Various motor-related brain regions are considered potential candidates for neuroimaging biomarkers associated with upper limb motor function after stroke [8–13]. The integrity of the corticospinal tract (CST), as assessed by diffusion tensor imaging (DTI), is considered one of the most important factors for motor function after stroke [8–10]. A study reported that low fractional anisotropy (FA) values in the ipsilesional CST were associated with poor motor function after stroke [9, 14]. In addition, motor recovery seemed to be associated with not only the integrity of CST but also the integrity of intra- and interhemispheric connections that may compensate for CST damage. Previous studies have investigated structural and functional alterations of intrahemispheric corticocortical connections between primary and secondary motor areas after stroke [13, 15–17]. In particular, FA values of intrahemispheric connections between the primary motor cortex (M1) and the ventral premotor cortex (PMv) were closely related to motor function in patients with relevant damage to the CST with severe motor deficits after stroke [15, 16]. The functional connectivity between the ipsilesional M1 and ipsilesional PMv was positively associated with motor function [12]. Furthermore, the functional connectivity of interhemispheric connections between the ipsilesional M1 and contralesional M1, which are connected by the corpus callosum (CC), was related to the integrity of CST in patients diagnosed with stroke [18]. Moreover, a cross-sectional study demonstrated that low FA values in CC were associated with poor motor function after stroke [19].

Brain-derived neurotrophic factor (BDNF), another potential prognostic factor after stroke, is an important neurotrophin that promotes axonal growth in neuroplasticity [20]. A common single-nucleotide polymorphism (SNP) in *BDNF*, i.e., a methionine (Met) substitution for valine (Val) at codon 66 (Val66Met; rs6265), is the most frequently investigated *BDNF* SNP [21]. The effects of the *BDNF* Val66Met polymorphism on neuroplasticity in motor recovery after stroke have been described in animals [22, 23]. In patients with ischemic stroke [5, 24, 25] and subarachnoid hemorrhage [26], the *BDNF* genotype has been associated with motor function. Furthermore, patients with stroke with the *BDNF* Val66Met polymorphism showed decreased brain activation of the ipsilesional primary sensorimotor cortex during affected hand movement compared with those without the *BDNF* polymorphism [27]. Despite the potential role of *BDNF* polymorphisms in neuroplasticity and motor function, only one study has previously demonstrated the relationship between CST integrity and motor function according to the *BDNF* genotype in patients with stroke [28]. Kim et al. [28] reported no differences in FA values of the ipsilesional CST between *BDNF* genotypes at 3 months after stroke. However, the relationships between the integrity of other motor-related white matter connections, such as intrahemispheric M1–PMv and interhemispheric M1–M1, as well as motor recovery according to the *BDNF* genotype, have not yet been elucidated.

The aims of this study were to use FA to assess the functional role of three motor-related white matter tracts according to the *BDNF* genotype in motor recovery in subacute stroke: the first consists of the majority of fibers from M1 to the medulla oblongata (CST), the second is the intrahemispheric connection from M1 to the ventral premotor cortex (M1PMv), and the third is the interhemispheric connection between bilateral M1s (CC). For each *BDNF* genotype, we investigated the relationship between motor impairment and tract-related FA of these white matter tracts. In particular, the *BDNF* genotype, which is related to brain plasticity and motor recovery, might play a role in the integrity of intra- and interhemispheric connections, which would compensate for CST damage.

## 2. Methods

### 2.1. Participants and Clinical Assessments

This was a retrospective study of patients with ischemic stroke who received intensive inpatient rehabilitation during the subacute phase after the onset of stroke. We enrolled patients aged 18–80 years who were diagnosed with first-time unilateral ischemic infarction with damage to the supratentorial area confirmed by brain magnetic resonance imaging. Patients with any unstable medical condition or neuropsychiatric comorbidities were excluded. Overall, 58 patients (19 females, 39 males; aged 62.9 ± 11.4 years) participated in the study. Motor outcomes and DTI results were assessed at 2 weeks (T1, 11.9 ± 2.5 days) and 3 months (T2, 92.9 ± 7.3 days) after stroke onset. Motor outcome was assessed using scores from the Fugl-Meyer assessment, upper extremity (FMA-UE; range: 0–66). Blood sampling for *BDNF* genotyping and assessment of MEP responses were conducted at T1. All patients received the same dose of physical and occupational therapy (3-week intensive inpatient rehabilitation).

Patients were divided into two groups according to the presence or absence of the *BDNF* Val66Met polymorphism: the Val group (Val/Val homozygotes) and the Met group (Met allele carriers—Val/Met heterozygotes or Met/Met homozygotes).

Written informed consent was obtained from all participants, and ethics approval was granted by the Institutional Review Board of Samsung Medical Center (IRB number 2018-04-146, clinical trial number NCT03647787).

### 2.2. Diffusion Tensor Imaging Data Acquisition

DTI data were acquired on the same day as clinical assessments using a Philips ACHIEVA MR scanner (Philips Medical Systems, Best, the Netherlands) operating at 3.0 Tesla. For each patient, 46 images were acquired with a single-shot, diffusion-weighted echo planar imaging sequence. Seventy-five axial slices were obtained that covered the entire brain with gradients (*b* = 1,000 mm^2^/s) applied along 45 noncollinear directions with the following sequence parameters: repetition time = 8,770 ms, echo time = 60 ms, field of view = 220 × 220 mm, slice thickness = 2.25 mm, and in-plane resolution 1.96 mm × 1.96 mm.

### 2.3. Preprocessing

The diffusion-weighted and anatomical images were analyzed using the FSL software package 5.1 (http://www.fmrib.ox.ac.uk/fsl). For each patient, the 46 images were initially realigned to the first image to correct for eddy current-induced distortions and simple head motions. The FA map was calculated at each voxel by fitting the diffusion tensor model. Each individual FA map was then registered nonlinearly to the FMRIB58_FA_1 mm standard space. During the registration process, stroke lesions were manually masked and excluded. After nonlinear coregistration to the FA map, nonlinear transformation was performed with anatomical images.

### 2.4. Probabilistic Tractography and Measurements of Tract-Related Fractional Anisotropy

Normalized and binarized template mask tracts (CST, M1PMv, and CC) in 26 healthy controls were used as templates that were developed based on probabilistic tractography as reported by Schulz et al. [16, 17, 29].

**Figure 1:**
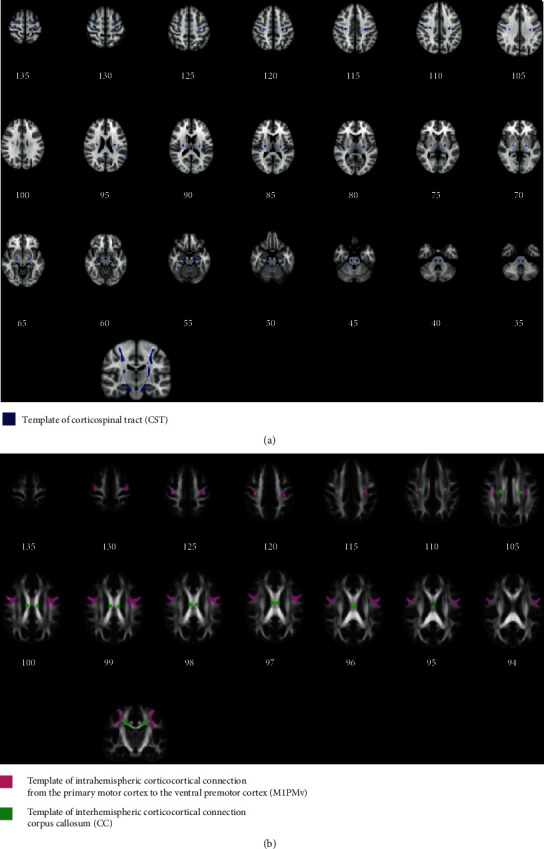
Template tract. Three template tracts were conducted by probabilistic tractography in 26 healthy subjects. The longitudinal tracts are shown in (a); the blue template is the corticospinal tract. The corticocortical connections are shown in (b); the pink template is intrahemispheric corticocortical connections from the primary motor cortex to the ventral premotor cortex, and the green template is interhemispheric corticocortical connections from both primary motor cortices.

To track the CST, individual seed masks for each hemisphere were placed in the hand knob area of M1 (MNI coordinates (37, −25, 62) and (−37, −25, 62)) for each patient using an established semiautomated pipeline. The target mask was the basis pontis. The waypoint masks included the posterior limb of the internal capsules and cerebral peduncles. For the CST, exclusion masks covering trajectories at the tegmentum pontis were added to the midsagittal, basal ganglia, and cerebellar regions. A total of 50,000 streamlines were sent from M1 to the spinal target masks in the ventral medulla oblongata. The CST output distributions were applied at three thresholds: 0.5%, 1%, and 2%. For each of the three thresholds, the common group average for each tract was calculated by summing all individual threshold- and subject-specific trajectories (Figure 1(a)).

To track the M1PMv, the seed mask was M1, and the target mask was the ventral premotor cortex; these were positioned at MNI coordinates (57, 14, 21) and (−57, 14, 21), respectively. To guide this first step of the tract reconstruction, interhemispheric and subcortical exclusion masks were used. A total of 100,000 streamlines were sent bidirectionally from both the seed and target regions. The five different thresholds (1%, 2%, 5%, 10%, and 20%) were applied to the final output. For each of the five thresholds, the common group average for each tract was calculated by taking the total of all individual threshold- and subject-specific trajectories (Figure 1(b)).

To track the CC, the left M1 was used as a seed mask, and the right M1 was used as a target mask. The waypoint mask, which was obtained from an atlas for subcortical segmentation in FreeSurferSeg (https://surfer.nmr.mgh.harvard.edu/), included the central portions of the CC. The exclusion masks were bilateral frontal and parietal lobes. A total of 100,000 streamlines were sent bidirectionally from both the seed and target regions. The five different thresholds (1%, 2%, 5%, 10%, and 20%) were applied to the final output. For each of the five thresholds, the common group average for each tract was calculated by summing all individual threshold- and subject-specific trajectories (Figure 1(b)).

These masks were used to calculate voxel-wise FA maps for the ipsilesional and contralesional tracts in patients with stroke. Absolute measures of the ipsilesional FA and contralesional FA were recorded, and the ratio of FA was defined as the ipsilesional FA/contralesional FA in CST and M1PMv, except for CC.

### 2.5. Statistical Analysis

All statistical analyses were performed using SPSS version 23.0 (SPSS Inc., Chicago, IL, USA). Assessments were determined to be normally distributed according to the Shapiro–Wilk test. We performed a *t*-test to compare the baseline characteristics of patients according to two *BDNF* genotype groups at T1. Furthermore, repeated measure analysis of variance with Bonferroni post hoc test was performed to evaluate the interaction effects of time (T1 and T2) and group (Val and Met) in the values of the ipsilesional FA, contralesional FA, and FA ratios in CST, M1PMv, and CC, which changed over time. Finally, Pearson correlation analyses were conducted between tract-related FA and FMA-UE at each time point according to each *BDNF* genotype. Partial correlation analyses were performed to assess the relationship between tract-related FA at T2 and FMA-UE at T2 according to the BDNF genotype. To adjust covariates in the partial correlation analyses, we analyzed Spearman's rank correlation between FMA-UE at T2 and clinical characteristics such as age and volume of stroke lesion at T1, FMA-UE at T1, and MEP response at T1 (*P* < 0.2). Age (rho = −0.212, *P* = 0.183), volume of stroke lesion (rho = −0.414, *P* = 0.098), and FMA-UE at T1 (rho = 0.873, *P* < 0.001) were used as covariates in the partial correlation analysis of T2. However, the MEP response at T1 was not used as a covariate because it was not associated with FMA-UE at T2 (rho = 0.082, *P* = 0.610).

## 3. Results

**Figure 2:**
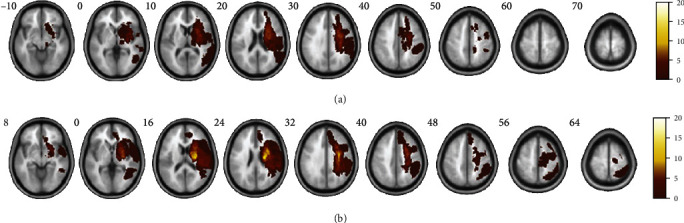
Individual lesion masks were drawn and then spatially normalized to the Montreal Neurological Institute (MNI) space. The decision to flip all left lesions to the right was arbitrary. The lesion masks of (a) valine (Val) homozygotes of the *BDNF* genotype and (b) methionine (Met) allele carriers of the *BDNF* genotype superimposed onto MNI space. Colored bars represent levels of overlap in each lesion. Red indicates infrequent overlap, while white indicates frequent overlap.

Table 1 and Supplementary Table S1 describe the general and stroke characteristics of the 58 patients. There were no significant differences in the distribution of sex, age, site of stroke lesion, type of stroke involvement, volume of stroke lesion (Figure 2), distribution of initial motor impairment severity, and FMA-UE scores at each time point.

**Figure 3:**
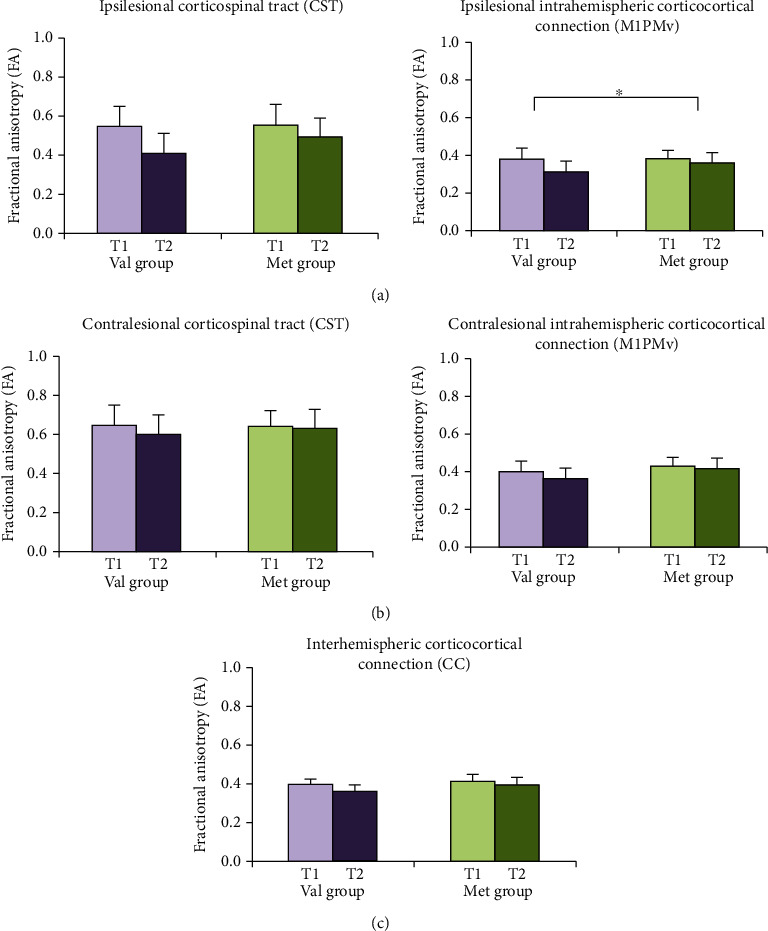
The absolute values of fractional anisotropy (FA) in the (a) ipsilesional, the (b) contralesional, and the (c) interhemispheric motor-related tracts between two weeks and three months after stroke in each *BDNF* genotype. CC: corpus callosum; CST: corticospinal tract; M1PMv: intrahemispheric corticocortical connection from the primary motor cortex to the ventral premotor cortex; Met group: *BDNF* methionine (Met) allele carriers; T1: two weeks after stroke onset; T2: three months after stroke onset; Val group: *BDNF* valine (Val) homozygotes. ^∗^*P* < 0.05.

There were changes in FA values from T1 to T2 in each BDNF group (Figure 3 and Supplementary Table S2). When comparing the interaction effect of time and BDNF genotype, there was a significant time and BDNF genotype interaction effect of FA in the ipsilesional M1PMv (*F* = 5.292, *P* = 0.025). In Bonferroni post hoc analysis, there was no significant difference in FA values in the ipsilesional M1PMv at T1 between the two groups (*F* = 0.398, *P* = 0.531); however, the FA values in the ipsilesional M1PMv at T2 in the Val group were significantly reduced compared with those in the Met group (*F* = 4.462, *P* = 0.039).

**Figure 4:**
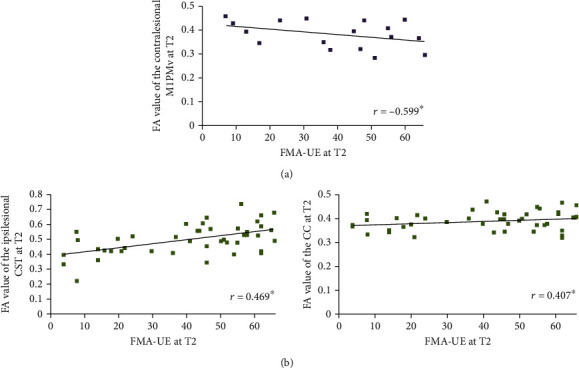
Correlations between upper limb motor function at three months after stroke and tract-related fractional anisotropy at three months after stroke in (a) valine (Val) homozygotes and (b) methionine (Met) allele carriers of *BDNF* genotype. “*r*” represents the partial correlation coefficient. Asterisks indicate significant differences (^∗^*P* < 0.05). CC: corpus callosum; CST: corticospinal tract; FA: fractional anisotropy; FMA-UE: Fugl-Meyer assessment, upper extremity; M1PMv: intrahemispheric corticocortical connection from the primary motor cortex to the ventral premotor cortex; *r*: partial correlation coefficient; T1: two weeks after stroke onset; T2: three months after stroke onset.

Pearson correlation analyses were conducted between tract-related FA and FMA-UE at each time point according to each *BDNF* genotype (Supplementary Tables S3 and S4). Partial correlation analyses were performed between FMA-UE at T2 and tract-related FA at T2 adjusted for age, volume of stroke lesion, and initial motor impairment at T1. In the Val group, FMA-UE at T2 was negatively correlated with FA in the contralesional M1PMv at T2 (*r* = −0.599, *P* = 0.024, 95% confidence interval (CI): 0.199–0.828). In the Met group, FMA-UE at T2 was positively correlated with FA in the ipsilesional CST and FA in the CC at T2 (*r* = 0.469, *P* = 0.003, and CI: 0.196–0.674; *r* = 0.407, *P* = 0.011, and CI: 0.121–0.630, respectively) (Figure 4 and Table 2).

## 4. Discussion

In this study, differential associations between upper limb motor impairment and tract-related FA were shown to be dependent on the *BDNF* genotype at the subacute phase after stroke. Our results indicate that the CST, M1PMv, and CC tracts exhibited decreased FA over time in both the Val homozygotes and Met allele carriers when considering the *BDNF* genotype. In addition, we demonstrated that FA values in the ipsilesional M1PMv of patients with Val homozygotes were more reduced compared with that in the Met group from 2 weeks to 3 months after stroke. However, the reason for this remains unclear. Reduction of FA values reflects a loss of integrity of the axolemma and/or myelin sheath along the white tract [30]. The FA value is also an indicator of the orientation of diffusion and could increase as a result of restricted perpendicular diffusivity, facilitated parallel diffusivity, or some combination of the two. When an axon fiber is branching and has a large diameter during the recovery stage from demyelination and/or axonal loss during the subacute phase after stroke, decreased FA values could be exhibited.

In Val homozygotes, upper limb motor impairment was negatively associated with FA of the contralesional M1PMv. Additionally, in Met allele carriers, motor impairment was positively associated with FA of the ipsilesional CST and CC. Integrity in the CST, M1PMv, and CC connections might be differentially related to motor impairment according to the *BDNF* genotype. However, it is unclear why white matter integrity of these tracts differentially links motor impairment at the subacute phase after stroke in each *BDNF* genotype. In the Met allele carriers, integrity of the ipsilesional CST and the interhemispheric connection were positively correlated with upper limb motor function. Furthermore, in the Val homozygotes, integrity of the contralesional intrahemispheric connection was negatively correlated with upper limb motor impairment. Considering these findings, one possible explanation is that the activity-dependent BDNF protein might influence white matter architecture. Met allele carriers might have redundant connectivity and endure microstructural damage after stroke. Indeed, Ziegler et al. [31] reported larger increases in healthy individuals in connections corresponding to corticospinal and interhemispheric connections among Met allele carriers than among Val homozygotes. In Met allele carriers, if silent axons are less likely to be pruned due to reduced BDNF secretion, structural connectivity might be less profoundly shaped by stroke than in Val homozygotes [31, 32].

Another possible interpretation is that the activity-dependent BDNF protein might be influential in the imbalance of interhemispheric inhibition after stroke. Previous studies reported a reduction of excitability in the ipsilesional hemisphere and an increase in excitability in the contralesional hemisphere after stroke [33, 34]. More contralesional excitability would be associated with less ipsilesional inhibition that is projected back to the contralesional hemisphere [35]. Patients with severe impairment retain abnormal contralesional activation, whereas those that exhibit substantial recovery over time show a normalization of brain activity to activation predominantly of the ipsilesional hemisphere [36, 37]. In Val homozygotes, as brain activity increases in the contralesional hemisphere, the activity-dependent BDNF protein might be more activated in the contralesional hemisphere, thereby interrupting motor recovery, compared with that in Met allele carriers. In contrast, in Met allele carriers, the activity-dependent BDNF protein in the contralesional hemisphere may remain at a similar level after stroke. As a result, Met allele carriers might be less affected by imbalances of interhemispheric inhibition. However, DTI does not provide information about the functional role of fibers, e.g., whether they are excitatory or inhibitory; thus, it will be necessary to explore functional and effective connectivity related to motor recovery after *BDNF* genotype-dependent stoke.

The present study had several limitations. First, the statistical power was too low to obtain a robust correlation between microstructural integrity and motor impairment in patients with Val homozygotes. To address this issue and provide sufficient statistical power, a larger sample number of Val homozygote patients with stroke should be evaluated; however, such individuals are underrepresented in East Asian populations. An international multicenter study is needed to offset the disparity in the *BDNF* genotype due to ethnicity. Second, correlation coefficients, which are used to assess the strength and direction of the linear relationships between pairs of variables, do not provide information about whether one variable moves in response to another [38]. Thus, to confirm causal relationships between motor function and tract-related FA according to the *BDNF* genotype following stroke, further study is needed using regression analysis, including DTI data before the stroke. Third, we did not consider serum levels of the BDNF protein, precursor BDNF, and BDNF propeptide, all of which would also affect brain neuroplasticity. A previous study reported that a combination therapy of rehabilitation and noninvasive brain stimulation in patients with stroke induced increased BDNF secretion and improved motor function [39]. Therefore, additional research is necessary to determine whether serum BDNF and precursor BDNF levels interact with motor function and the microstructural integrity of motor-related tracts according to the *BDNF* polymorphism. Finally, interpretation of FA values in terms of discrete pathological processes appears to be disputable. In analyses of white matter tract integrity, the issue of intravoxel crossing fibers may be particularly problematic.

In light of the above, the *BDNF* genotype could be considered as a factor that may influence functional recovery in patients with stroke. Our results may be useful for improving stroke recovery prediction models, especially for patients with upper extremity motor impairment for whom the proportional recovery rule after stroke does not apply.

## 5. Conclusions

The microstructural integrity of both the intra- and interhemispheric connections is differentially related to motor recovery based on the specific *BDNF* genotype in patients with stroke. Thus, the *BDNF* genotype could be considered as a factor that may influence neuroplasticity, reorganization, and the functional role of white matter tracts related to motor recovery following stroke.

## Figures and Tables

**Table 1:** Characteristics of participants according to *BDNF* genotype.

	Groups		*P* value
	Val (*N* = 17)	Met (*N* = 41)	
Female, *N* (%)	6 (35.3)	13 (31.7)	0.767
Age, mean ± SD (range) (yr)	64.7 ± 12.4 (35-80)	62.1 ± 11.7 (33-80)	0.492
Stroke lesion type, *N*			
Right : left hemisphere	10 : 7	20 : 21	0.777
Cortical : subcortical : corticosubcortical lesion	1 : 12 : 4	7 : 31 : 3	0.557
Volume of stroke lesion (cm^3^)	17.2 ± 16.9	10.3 ± 12.2	0.082
FMA-UE scores at T1, mean ± SD (range)	26.6 ± 21.8 (0-62)	28.3 ± 16.5 (0-61)	0.750
FMA-UE scores at T2, mean ± SD (range)	39.2 ± 19.5 (7-66)	42.1 ± 19.2 (4-66)	0.599
Absence of MEP response at T1, *N* (%)	10 (58.8)	26 (63.4)	0.773

*N*: the number of patients; mean ± SD: mean ± standard deviation; *BDNF*: brain-derived neurotrophic factor; FMA-UE: Fugl-Meyer assessment, upper extremity score; MEP: motor evoked potential; Met: carriers of methionine allele of *BDNF* genotype; T1: two weeks after stroke onset; T2: three months after stroke onset; Val: patients with valine homozygotes of *BDNF* genotype.

**Table 2:** Partial correlation adjusted by age, stroke lesion volume, and severity of initial motor impairments between tract-fractional anisotropy at T2 and upper limb motor impairments at T2 in patients with the valine homozygotes of the *BDNF* genotype (Val group) and methionine allele carriers of *BDNF* genotype (Met group).

		Val group	Met group
Tracts		FMA-UE at T2	FMA-UE at T2
CST	Ipsilesional	0.338	0.469^∗^
	Contralesional	-0.382	0.276
	Ratio	0.254	0.259
M1PMv	Ipsilesional	-0.388	0.286
	Contralesional	-0.599^∗^	0.092
	Ratio	0.134	0.228
CC		-0.372	0.407^∗^

Each cell represents correlation coefficients (*r*). FMA-UE: Fugl-Myer assessment, upper extremity score; CC: corpus callosum; CST: corticospinal tract; FA: fractional anisotropy; M1PMv: intrahemispheric corticocortical connection from primary motor cortex to ventral premotor cortex; T1: two weeks after stroke onset; T2: three months after stroke onset; ^∗^*P* < 0.05, ^∗∗^*P* < 0.001.

## Data Availability

The data are in the supplementary information files.

## Supplementary Materials

Supplementary Materials Supplementary Table S1: characteristics of participants. Supplementary Table S2: the values of tract-related fractional anisotropy (FA) in patients with valine homozygote (Val) and methionine allele carriers (Met) of the *BDNF* genotype. Supplementary Table S3: correlation between the value of tract-related fractional anisotropy and the Fugl-Meyer assessment; upper extremity score at each time point in patients with the valine homozygote of the *BDNF* genotype. Supplementary Table S4: correlation between the value of tract-related fractional anisotropy and the Fugl-Meyer assessment; upper extremity score at each time point in patients with methionine allele carriers of the *BDNF* genotype.
