# Supplementary material for: Differential Relationship between Microstructural Integrity in White Matter Tracts and Motor Recovery following Stroke Based on Brain-Derived Neurotrophic Factor Genotype
Source: Neural Plast. 2020 Sep 22;2020:5742421. doi: 10.1155/2020/5742421 (PMC7527931; doi:10.1155/2020/5742421)
Supplement: Supplementary Materials — Supplementary Table S1: characteristics of participants. Supplementary Table S2: the values of tract-related fractional anisotropy (FA) in patients with valine homozygote (Val) and methionine allele carriers (Met) of the BDNF genotype. Supplementary Table S3: correlation between the value of tract-related fractional anisotropy and the Fugl-Meyer assessment; upper extremity score at each time point in patients with the valine homozygote of the BDNF genotype. Supplementary Table S4: correlation between the value of tract-related fractional anisotropy and the Fugl-Meyer assessment; upper extremity score at each time point in patients with methionine allele carriers of the BDNF genotype. [file 5742421.f1.docx]

**Table S1.** Characteristics of Participants.

| No. | Sex | Age | BDNF genotype | Onset | Stroke lesion | | | | FMA-UE | | |
| --- | --- | --- | --- | --- | --- | --- | --- | --- | --- | --- | --- |
|  |  |  |  |  | Site | Location | Territory | Volume (cm^3^) | | T1 | T2 |
| 1 | M | 33 | Val/Met | 2009-12-25 | Lt. | CR | MCA | 1.06 | | 16 | 37 |
| 2 | F | 69 | Val/Met | 2010-03-12 | Lt. | frontal cortex | ACA | 6.52 | | 17 | 40 |
| 3 | F | 67 | Val/Met | 2010-04-13 | Rt. | SC | MCA | 2.18 | | 18 | 18 |
| 4 | F | 52 | Val/Met | 2010-05-05 | Rt. | SC | MCA | 3.91 | | 16 | 16 |
| 5 | M | 40 | Val/Met | 2010-07-22 | Rt. | insular cortex; M2, ,M3 | MCA | 21.5 | | 28 | 50 |
| 6 | F | 58 | Val/Met | 2010-09-10 | Lt. | CR/SC/PLIC | MCA | 12.15 | | 4 | 4 |
| 7 | M | 64 | Met/Met | 2010-11-01 | Lt. | CR | MCA | 16.42 | | 10 | 24 |
| 8 | M | 67 | Val/Val | 2011-04-05 | Rt. | CR/SC | MCA | 8.15 | | 43 | 65 |
| 9 | M | 57 | Val/Met | 2011-07-13 | Lt. | parietal cortex; M4 | MCA | 37.91 | | 24 | 45 |
| 10 | F | 76 | Val/Val | 2011-07-20 | Rt. | parietal cortex; M4 | MCA | 27.74 | | 0 | 14 |
| 11 | M | 63 | Val/Val | 2011-09-08 | Lt. | CR/SC | MCA | 35.7 | | 23 | 44 |
| 12 | M | 64 | Met/Met | 2011-10-10 | Rt. | CR/SC | MCA | 3.07 | | 32 | 46 |
| 13 | M | 72 | Met/Met | 2011-11-11 | Rt. | frontal, insular cortex; M2, M3, M4 | MCA | 38.56 | | 48 | 62 |
| 14 | M | 71 | Val/Val | 2011-12-11 | Lt. | SC | MCA | 2.54 | | 24 | 43 |
| 15 | M | 52 | Met/Met | 2011-12-19 | Lt. | CR | MCA | 4 | | 22 | 52 |
| 16 | M | 60 | Val/Met | 2012-01-04 | Lt. | PLIC | MCA | 2.02 | | 45 | 57 |
| 17 | F | 66 | Met/Met | 2012-02-02 | Rt. | CR, SC | MCA | 1.2 | | 31 | 46 |
| 18 | F | 74 | Met/Met | 2012-03-03 | Rt. | insular cortex; M2,M3, | MCA | 12.42 | | 12 | 20 |
| 19 | M | 63 | Val/Met | 2012-03-26 | Lt. | CR/SC | MCA | 6.55 | | 48 | 58 |
| 20 | M | 63 | Val/Val | 2012-04-02 | Lt. | SC | MCA | 3.23 | | 40 | 62 |
| 21 | M | 79 | Val/Met | 2012-06-07 | Rt. | frontal cortex; M4 | MCA | 6.7 | | 22 | 61 |
| 22 | M | 78 | Val/Val | 2012-06-11 | Rt. | ACA-MCA watershed | BDZ | 37.35 | | 9 | 8 |
| 23 | M | 80 | Val/Val | 2012-06-16 | Rt. | insular, parietal cortex; M2, M3, M4 | MCA | 32.77 | | 19 | 56 |
| 24 | M | 71 | Val/Val | 2012-07-06 | Lt. | CR | MCA | 1.32 | | 10 | 8 |
| 25 | M | 74 | Val/Met | 2012-07-10 | Lt. | CR | MCA | 2.36 | | 42 | 55 |
| 26 | M | 77 | Val/Met | 2012-07-12 | Lt. | CR | MCA | 1.47 | | 61 | 66 |
| 27 | M | 76 | Val/Met | 2012-10-04 | Lt. | frontal cortex; M4 | MCA | 1.24 | | 9 | 22 |
| 28 | M | 59 | Val/Met | 2012-11-14 | Lt. | insular cortex; M2 | MCA | 24.56 | | 27 | 61 |
| 29 | F | 76 | Val/Met | 2012-12-03 | Rt. | SC | MCA | 12.97 | | 30 | 51 |
| 30 | F | 48 | Val/Met | 2012-12-05 | Lt. | CR, SC | MCA | 17.99 | | 34 | 47 |
| 31 | M | 54 | Met/Met | 2012-12-22 | Lt. | CR, SC | MCA | 22.44 | | 52 | 62 |
| 32 | M | 78 | Val/Met | 2012-12-29 | Lt. | insular cortex; M2 | MCA | 1.82 | | 22 | 36 |
| 33 | M | 37 | Val/Met | 2013-02-03 | Rt. | CR | MCA | 2.54 | | 60 | 58 |
| 34 | M | 63 | Met/Met | 2013-02-10 | Rt. | TH | PCA | 2.54 | | 44 | 55 |
| 35 | M | 52 | Val/Val | 2013-02-23 | Rt. | SC, insular cortex; M1, M2 | MCA | 32.18 | | 16 | 30 |
| 36 | M | 61 | Met/Met | 2013-03-04 | Rt. | CR | MCA | 2.03 | | 24 | 21 |
| 37 | F | 49 | Val/Val | 2013-03-07 | Lt. | CR | MCA | 2.56 | | 55 | 66 |
| 38 | M | 61 | Val/Val | 2013-03-14 | Rt. | insular cortex; M2, M3 | MCA | 43.06 | | 56 | 62 |
| 39 | M | 51 | Val/Met | 2013-03-29 | Rt. | CR | MCA | 3.96 | | 18 | 41 |
| 40 | F | 70 | Val/Val | 2013-04-01 | Rt. | SC | MCA | 8.61 | | 41 | 54 |
| 41 | F | 54 | Met/Met | 2013-04-29 | Lt. | CR | MCA | 3.1 | | 4 | 4 |
| 42 | M | 63 | Val/Met | 2013-05-21 | Lt. | CR, SC | MCA | 4.01 | | 16 | 37 |
| 43 | F | 79 | Val/Met | 2013-06-08 | Rt. | frontal cortex | ACA | 20.95 | | 17 | 40 |
| 44 | M | 35 | Val/Val | 2013-09-23 | Rt. | CR | MCA | 3.12 | | 18 | 18 |
| 45 | M | 52 | Met/Met | 2013-10-19 | Rt. | CR/PLIC | MCA | 1.54 | | 16 | 16 |
| 46 | F | 53 | Val/Val | 2014-03-15 | Rt. | TH | PCA | 4.07 | | 28 | 50 |
| 47 | M | 75 | Met/Met | 2014-03-26 | Rt. | SC | MCA | 8.26 | | 4 | 4 |
| 48 | M | 66 | Val/Met | 2014-04-25 | Rt. | CR | MCA | 36.19 | | 10 | 24 |
| 49 | M | 54 | Val/Met | 2014-04-29 | Rt. | CR/PLIC | MCA | 49.39 | | 43 | 65 |
| 50 | M | 63 | Val/Met | 2014-06-15 | Lt. | CR | MCA | 4.44 | | 24 | 45 |
| 51 | F | 65 | Val/Met | 2014-08-30 | Rt. | CR | MCA | 2.39 | | 0 | 14 |
| 52 | M | 66 | Val/Val | 2014-08-30 | Lt. | frontal, insular cortex; M2, M3, M4 | MCA | 44.37 | | 23 | 44 |
| 53 | M | 55 | Met/Met | 2014-11-20 | Lt. | CR/SC | MCA | 5.76 | | 32 | 46 |
| 54 | F | 79 | Val/Val | 2015-01-10 | Rt. | CR | MCA | 4.03 | | 48 | 62 |
| 55 | F | 48 | Val/Met | 2015-01-19 | Rt. | CR | MCA | 1.43 | | 24 | 43 |
| 56 | F | 80 | Val/Met | 2015-04-29 | Lt. | CR | MCA | 1.01 | | 22 | 52 |
| 57 | M | 67 | Val/Met | 2015-05-05 | Lt. | CR/SC | MCA | 9.98 | | 45 | 57 |
| 58 | F | 73 | Val/Val | 2015-08-23 | Lt. | CR/SC | MCA | 2.11 | | 31 | 46 |

ACA: anterior cerebral artery; BDZ: borderzone; CR: corona radiata; F: female; FMA-UE: Fugl-Meyer assessment, upper extremity score; Lt.: left; M: male; M1: the sphenoidal segments of middle cerebral artery; M2: the insular segments of middle cerebral artery; M3: the opercular segments of middle cerebral artery; M4: the cortical segments of middle cerebral artery; MCA: middle cerebral artery; Met: methionine; PCA: posterior cerebral artery; PLIC: posterior limb of internal capsule; Rt.: right; SC: striatocapular area; T1: 2 weeks after stroke onset; T2: 3 months after stroke onset; TH: thalamus; Val: valine

**Table S2**. The values of tract-related fractional anisotropy (FA) in patients with Valine homozygote (Val) and Methionine allele carriers (Met) of *BDNF* Genotype.

|  | | Val | | | Met | | |
| --- | --- | --- | --- | --- | --- | --- | --- |
| Tracts |  | T1 | T2 |  | | T1 | T2 |
| CST | Ipsilesional | 0.53 ± 0.09 | 0.43 ± 0.09 |  | | 0.56 ± 0.09 | 0.51 ± 0.09 |
|  | Contralesional | 0.61 ± 0.09 | 0.58 ± 0.08 |  | | 0.64 ± 0.06 | 0.64 ± 0.08 |
|  | Ratio | 0.87 ± 0.11 | 0.73 ± 0.14 |  | | 0.88 ± 0.11 | 0.79 ± 0.12 |
| M1PMv | Ipsilesional | 0.38 ± 0.06 | 0.34 ± 0.05 |  | | 0.39 ± 0.05 | 0.37 ± 0.05 |
|  | Contralesional | 0.41 ± 0.04 | 0.38 ± 0.05 |  | | 0.43 ± 0.04 | 0.41 ± 0.04 |
|  | Ratio | 0.95 ± 0.12 | 0.93 ± 0.13 |  | | 0.92 ± 0.12 | 0.90 ± 0.12 |
| CC |  | 0.38 ± 0.04 | 0.36 ± 0.04 |  | | 0.41 ± 0.04 | 0.39 ± 0.04 |

Each cell represents mean ± standard deviation. CC: corpus callosum; CST: corticospinal tract; M1PMv: intrahemispheric corticocortical connection from primary motor cortex to ventral premotor cortex; T1: 2 weeks after stroke onset; T2: 3 months after stroke onset.

**Table S3.** Correlation between the value of tract-related fractional anisotropy and the Fugl-Meyer assessment, upper extremity score at each time point in patients with Valine homozygote of BDNF genotype.

| At T1 | Ipsilesional CST | Contralesional CST | CST ratio | Ipsilesional M1PMv | Contralesional M1PMv | M1PMv ratio | CC |
| --- | --- | --- | --- | --- | --- | --- | --- |
| FMA-UE | 0.311 | -0.578^*^ | 0.428 | -0.330 | 0.446 | -0.160 | 0.181 |
|  | | | | | | | |
| At T2 | Ipsilesional CST | Contralesional CST | CST ratio | Ipsilesional M1PMv | Contralesional M1PMv | M1PMv ratio | CC |
| FMA-UE | 0.308 | -0.582^*^ | 0.700^**^ | 0.275 | -0.428 | 0.252 | 0.102 |
|  | | | | | | | |
| From T1 to T2 | △Ipsilesional CST | △Contralesional CST | △CST ratio | △Ipsilesional M1PMv | △Contralesional M1PMv | △M1PMv ratio | △ CC |
| △FMA-UE | 0.172 | -0.244 | 0.273 | 0.128 | -0.236 | 0.326 | 0.273 |

Each cell represents correlation coefficients (r). CC: corpus callosum; CST: corticospinal tract; FMA-UE: Fugl-Meyer assessment, upper extremity score; M1PMv: intrahemispheric corticocortical connection from primary motor cortex to ventral premotor cortex; T1: two weeks after stroke onset; T2: three months after stroke onset. ^*^*P* < 0.05, ^**^*P* < 0.001.

**Table S4.** Correlation between the value of tract-related fractional anisotropy and the Fugl-Meyer assessment, upper extremity score at each time point in Methionine allele carriers of BDNF genotype.

| At T1 | Ipsilesional CST | Contralesional CST | CST ratio | Ipsilesional M1PMv | Contralesional M1PMv | M1PMv ratio | CC |
| --- | --- | --- | --- | --- | --- | --- | --- |
| FMA-UE | 0.307 | 0.260 | 0.196 | 0.190 | -0.183 | 0.209 | -0.212 |
|  | | | | | | | |
| At T2 | Ipsilesional CST | Contralesional CST | CST ratio | Ipsilesional M1PMv | Contralesional M1PMv | M1PMv ratio | CC |
| FMA-UE | 0.489^**^ | 0.254 | 0.356^*^ | 0.244 | -0.094 | 0.291 | 0.410^*^ |
|  | | | | | | | |
| From T1 to T2 | △Ipsilesional CST | △Contralesional CST | △CST ratio | △Ipsilesional M1PMv | △Contralesional M1PMv | △M1PMv ratio | △ CC |
| △FMA-UE | 0.241 | 0.281 | 0.231 | 0.214 | 0.092 | 0.014 | 0.308 |

Each cell represents correlation coefficients (r). CC: corpus callosum; CST: corticospinal tract; FMA-UE: Fugl-Meyer assessment, upper extremity score; M1PMv: intrahemispheric corticocortical connection from primary motor cortex to ventral premotor cortex; T1: two weeks after stroke onset; T2: three months after stroke onset. ^*^*P* < 0.05, ^**^*P* < 0.001.
